# Evaluation of the efficiency of cyclodextrin polymers as sustainable sampling material for catching palytoxin-like compounds in seawater

**DOI:** 10.1007/s00604-025-07507-0

**Published:** 2025-09-10

**Authors:** Antonella Miglione, Chiara Melchiorre, Samuela Capellacci, Luciana Tartaglione, Michela Varra, Alex Fragoso, Silvia Casabianca, Mabel Torréns, Jorge Diogène, Antonella Penna, Carmela Dell’Aversano, Mònica Campàs

**Affiliations:** 1 Department of Pharmacy, University of Naples Federico II, School of Medicine and Surgery, Via D. Montesano 49, 80131 Naples, Italy; 2 NBFC, National Biodiversity Future Center, 90133 Palermo, Italy; 3 Department of Biomolecular Sciences, University of Urbino, Campus E. Mattei, 61029 Urbino, Italy; 4 Departament d’Enginyeria Química, Universitat Rovira i Virgili, Av. Països Catalans 26, 43007 Tarragona, Spain; 5 Marine and Continental Waters, IRTA, Ctra. Poble Nou km 5.5, 43540 La Ràpita, Spain

**Keywords:** Cyclodextrins, Palytoxins, Ovatoxins, *Ostreopsis* cf. *ovata*, LC–MS, Passive sampling

## Abstract

Palytoxin-like compounds, including ovatoxins, are potent emerging toxins responsible for human respiratory poisonings following inhalation of contaminated marine aerosols. Periodic massive proliferations of the ovatoxin-producing organism (*Ostreopsis* cf. *ovata*) worldwide, particularly in the Mediterranean, have caused severe toxic outbreaks, drawing the attention of health authorities. At present, an efficient and sustainable sampling system for monitoring ovatoxins in seawater remains unavailable. Herein, different cyclodextrin (CD) polymers were investigated as a green and effective alternative to conventional and low-performing resins to detect ovatoxins in seawater. Spiking experiments using different concentrations of palytoxin or ovatoxins (namely 200 and 3.3 ng PLTX/mL or 200 ng OVTX-a/mL) were conducted and LC-HRMS was used to evaluate the suitability of CD polymers in capturing palytoxin-like compounds. Several conditions were tested for extracting polymer materials, including different extraction times (1.5 to 4 h), various solvent mixtures (acidic or alkaline), and organic modifiers (methanol or acetonitrile) at different ratios. Among the tested polymers, γ-CD-hexamethylene diisocyanate (HDI) resulted to be the most promising one, providing ovatoxin recoveries in the range 82–108% at a spiking level of 200 ng OVTX-a per mL. The best extracting condition was alkaline pH methanol:water 8:2 mixture, which showed the best palytoxin recovery in both high and low concentration spiking experiments. Finally, a time-dependent increase in the amount of ovatoxins captured by γ-CD-HDI disks deployed in *O*. cf. *ovata* cultures was observed. These findings provide valuable insights on the efficiency of passive sampling using CD polymers for capturing ovatoxins during *O*. cf. *ovata* bloom events.

**Graphical Abstract:**

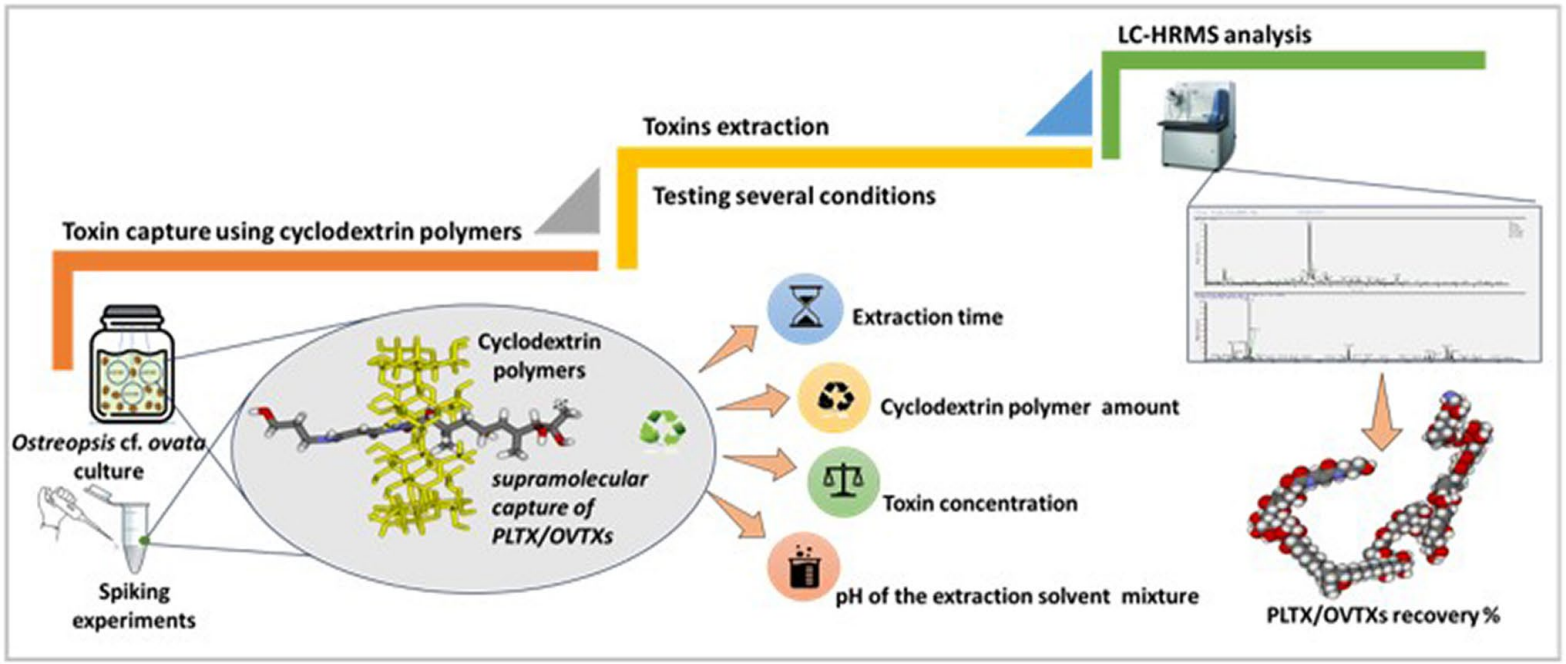

**Supplementary Information:**

The online version contains supplementary material available at 10.1007/s00604-025-07507-0.

## Introduction

**Fig. 1:**
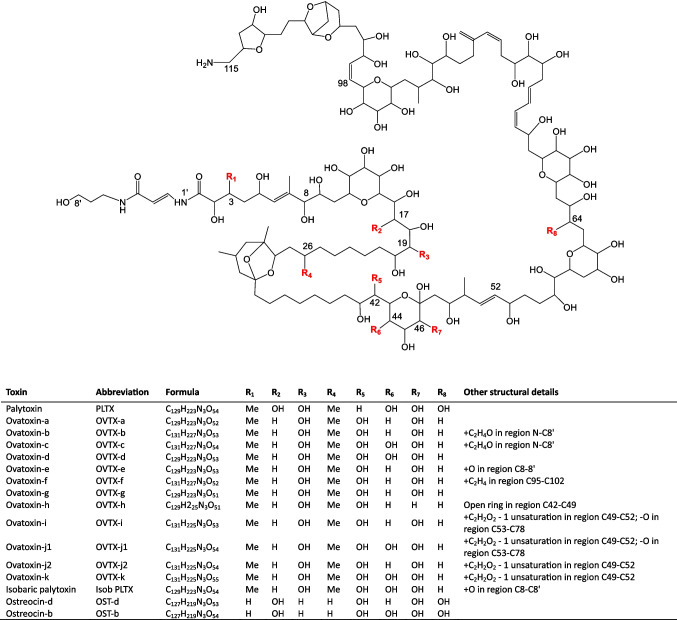
Planar structures of palytoxin (PLTX) and some of its analogues from *Ostreopsis* spp*.* Structures of PLTX, ostreocin-d (OST-d), and ovatoxin-a (OVTX-a) were elucidated by NMR, while structures of other congeners were proposed based on LC-HRMS^n^ (n = 1, 2, and/or 3) data only. Figure modified from Miele et al. [5]

Palytoxin (PLTX) and its structural analogues, including ovatoxins (OVTXs) and ostreocins (OSTs), belong to a family of over 30 complex and fascinating marine neurotoxins currently considered among the most potent non-proteinaceous natural compounds so far known (Fig. 1) [1–4].

Despite PLTX was firstly identified in soft zoanthid corals (*Palythoa* spp*.*), the natural origin of PLTX-like compounds has long been debated with different postulated production sources spanning from benthic dinoflagellates (*Ostreopsis* spp.) to cyanobacteria (*Trichodesmium*) [1, 6–8]. Among these, *Ostreopsis fattorussoi* and *Ostreopsis* cf. *ovata* are the most widely recognized producers of OVTXs while *Ostreopsis siamensis* is regarded as producer of OSTs [9–11].

Even more intriguing are the adverse effects associated with oral, dermal, and inhalation/ocular exposure to PLTX-like compounds in humans [9, 12–15]. Interestingly, different toxic effects have been observed depending on geographical locations and the specific toxin-producing organisms involved [16–23]. Respiratory syndromes are the predominant outcome in temperate regions, with hundreds of cases of dermatitis and/or respiratory symptoms reported in people exposed to toxic aerosols during *O*. cf. *ovata* blooms, with the effects attributed to OVTXs [9, 24–26].

Recently, Ternon et al. [27] have investigated the aerosolization of *Ostreopsis* toxins by analyzing both the dissolved and particulate fractions of seawater to identify the sources of aerosolized toxins highlighting that solubilized ovatoxins in natural seawater appear to be present only in very low amounts [28, 29]. However, discrepancies between laboratory experiments—showing enhanced aerosolization of ovatoxin-a at low cell concentrations (< 10^6^ cells/L)—and field observations, where the presence of OVTXs and *Ostreopsis* cell debris in sea spray aerosols has been associated with higher cell concentrations (> 10^6^ cells/L) during the stationary phase of blooms [30], have hindered a comprehensive understanding of the aerosolization process. This emphasizes the need for in-depth investigation of toxin concentrations within the water column, an aspect often overlooked in most previous studies that have primarily focused on the toxin profiles of cells inhabiting the benthic compartment [31–36]. These uncertainties in the aerosolization mechanisms, combined with the lack of ecotoxicological data, complicate the assessment of public health risks. Moreover, public awareness about *Ostreopsis* spp. associated risks is currently scarce among citizens, including coastal residents and tourists. Nonetheless, the impact of PLTX-like compounds on human health, which becomes increasingly evident as research progresses, has prompted national agencies and the European Food Safety Authority to clearly express the need for managing the *Ostreopsis* phenomenon through surveillance activities and monitoring programmes [37–39]. Currently, monitoring programmes of *O*. cf. *ovata* are conducted through cell counts on macroalgae and in the water column, with sampling typically carried out every 2 weeks [40]. This approach requires considerable financial resources, is labor-intensive, and demands frequent fieldwork as well as laboratory analyses, often involving multiple operators and the transport of large volumes of seawater, making it both time-consuming and logistically challenging. Moreover, this approach does not account for the biological variability in toxin production among different *O*. cf. *ovata* strains.

In such a perspective, the development of suitable time-integrated passive sampling methods cost-effective, easy to deploy and recover, offers a promising alternative allowing for the collection of toxins from seawater over time, during *O*. cf. *ovata* blooms. This approach supports large-scale monitoring programmes without the need for continuous time-consuming sampling efforts. Such a sampling strategy could identify potential intoxication risks even when highly toxin-producing strains are present in the seawater at low cell abundances. Therefore, this kind of sampling methods represent a valuable approach for assessing toxin levels and trends in affected areas, thus improving public health protection. Unfortunately, previously reported attempts to use the most commonly employed polymeric and lipophilic resins such as HP-20, HLB, or STRATA X for passive sampling of OVTXs in seawater [41–43] were not successful, resulting in poor toxin recoveries. This highlights the challenges associated with effectively capturing these compounds from seawater using conventional sorbent materials. In addition, active carbon-based sorbent materials have been shown to irreversibly bind PLTX, making them unsuitable for the detection of PLTX-like compounds in seawater [44]. Worsening the situation, PLTX-like compounds are tricky molecules to work with. Their structure, characterized by numerous hydroxyl groups, unsaturation, amines, amides, cyclic ethers, ketal/hemiketal functionalities, and a long continuous chain of carbon atoms, confers both lipophilic and hydrophilic properties [45]. This structural complexity poses significant challenges in the handling and detection of PLTX-like compounds [5, 46] and hinders the correct and accurate determination of these toxins in environmental and seafood matrices.

**Fig. 2:**
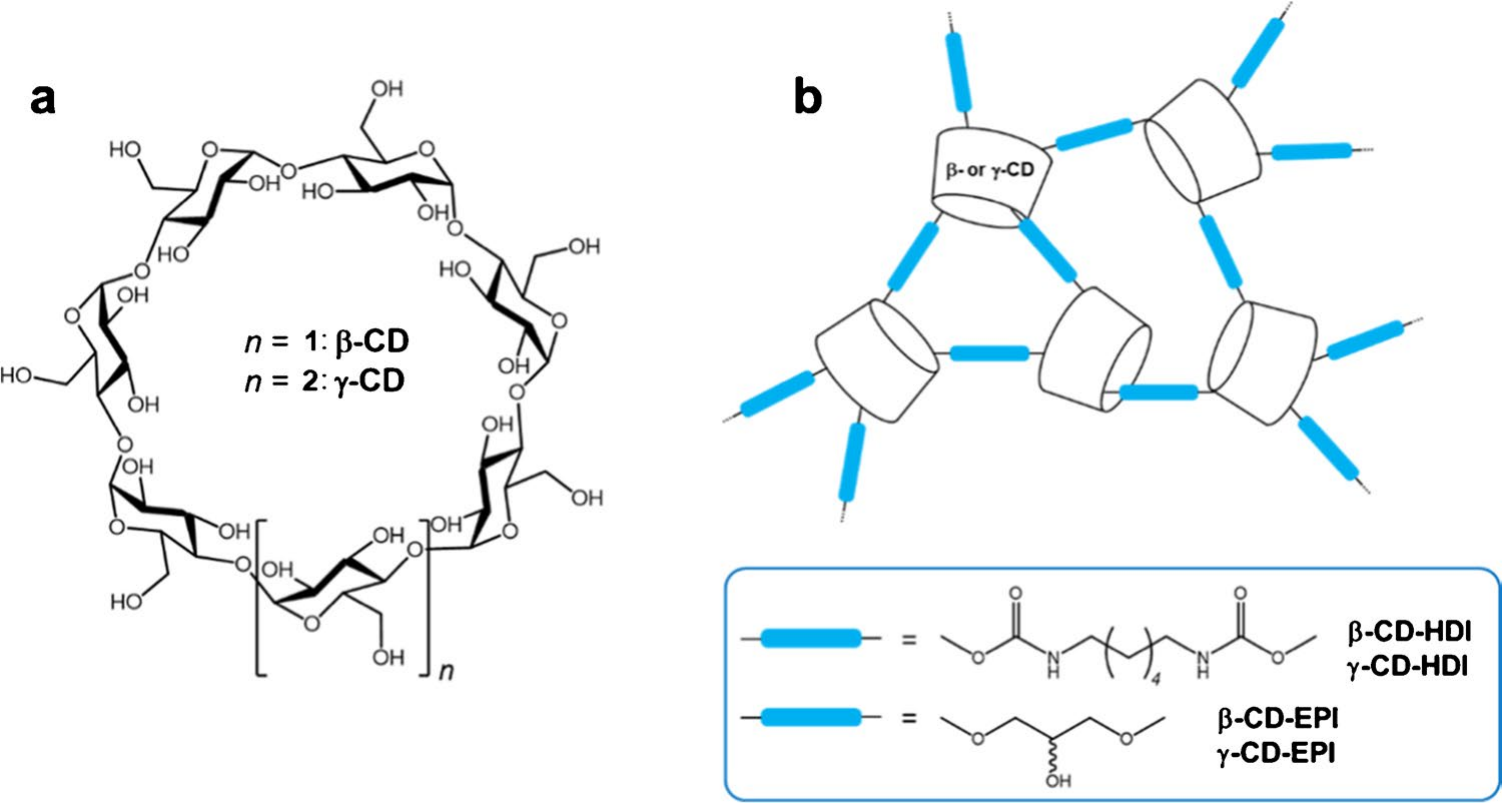
**a** Structure of β- and γ-cyclodextrins (β/γ-CD). **b** Schematic representations β/γ-CD-hexamethylene diisocyanate (HDI) and β/γ-CD-epichlorohydrin (EPI) polymers

Supramolecular chemistry has been extensively studied for applications in adsorption and separation employing macrocyclic structures [47–49]. The dynamic and reversible nature of non-covalent interactions offers a good alternative to enhance the affinity, kinetics, and saturation behavior of sorbent materials [50]. Among the several classes of macrocyclic compounds, cyclodextrins (CDs) constitute a family of cyclic α−1 → 4-linked glucose oligomers (Fig. 2a) possessing a truncated cone structure with a hydrophobic internal cavity able to form inclusion complexes with a wide variety of organic compounds [51]. This may result in different binding affinities, improved selectivity, and enhanced efficiency in capturing a wide variety of target compounds [52–54].

CD polymers have been successfully used for passive sampling of lipophilic toxins [55] and their use in clean-up procedures has also been recently demonstrated [56, 57]. Based on these encouraging data reported in the literature, this study investigates CD polymers bearing hydrophobic or hydrophilic cross-linkers (Fig. 2b) for the capture of amphiphilic toxins (PLTXs) as a sustainable alternative to conventional resins such as commercial Diaion® HP-20 (used as a control), with potential application in environmental monitoring of OVTXs. To this scope, different CD polymers were first tested in toxin-free seawater spiked with a known amount of PLTX standard or OVTXs in *O.* cf*. ovata* crude extract and the toxin recovery, after each CD polymer extraction, was evaluated by LC-HRMS analysis. Subsequently, the potential to develop an early warning tool that could promptly capture PLTX-like compounds in the sea, thus detecting *O.* cf. *ovata* proliferation, was explored by deploying CD-based passive sampling disks into *O*. cf. *ovata* cultures.

## Experimental section

### Reagents

Organic solvents (HPLC grade ≥ 99.9%)—methanol (MeOH), ethanol (EtOH), and acetonitrile (ACN)—were from VWR Chemicals Srl (Milan, Italy). Laboratory grade trifluoroacetic acid (TFA), acetic acid (AA), formic acid (FA), ammonium acetate (AcNH_4_), sodium hydroxide (NaOH), and water (HPLC grade) were from Sigma Aldrich (Darmstadt, Germany). Milli-Q water was produced in-house to 18 MΩ × cm quality, using a Milli-Q integral 3 system from Merck Millipore KGaA (Darmstadt, Germany).

A non-certified standard of PLTX (100 μg) from Wako Chemicals GmbH (Neuss, Germany) was dissolved in 10 mL of 50% aqueous EtOH and used as stock solution (10 μg/mL) in all the experiments.

Toxin-free (blank) natural seawater was collected from coastal areas near Naples, filtered through Durapore® 0.45 μm membrane to remove particulate organic matter, analyzed by LC-HRMS to confirm the absence of PLTXs and OVTXs, and stored at − 20 °C before use as described in previous study [44].

### Cyclodextrin polymer synthesis and disk preparation

The following CD polymers were tested: β-cyclodextrin-hexamethylene diisocyanate (β-CD-HDI), β-cyclodextrin-epichlorohydrin (β-CD-EPI), γ-cyclodextrin-hexamethylene diisocyanate (γ-CD-HDI), and γ-cyclodextrin-epichlorohydrin (γ-CD-EPI). The HDI polymers were synthesized by reaction of the native dried CDs with hexamethylene diisocyanate (1:7 and 1:8 M ratio, respectively) in dimethylformamide containing triethylamine [58]. Meanwhile, the EPI polymers were prepared by reaction of the native CDs with epichlorohydrin (1:14 and 1:16 molar ratio, respectively) in concentrated NaOH [59]. The crude materials were purified by extensive Soxhlet extraction with EtOH and water. Diaion® HP-20 Supelco resin was obtained from VidraFoc (Barcelona, Spain). Passive sampling disks were constructed by placing 1 g of γ-CD-HDI or Diaion® HP-20 between two layers of 1-μm nylon mesh (Sefar Maissa S.A.U., Cardedeu, Barcelona, Spain), clipped between two cylindrical PVC rings (4-cm diameter for immersion in cultures) (Fig. S1). CD polymers and Diaion® HP-20 resin were activated before each experiment, by soaking the disks in MeOH for 15 min and rinsing with Milli-Q water.

### *Ostreopsis *cf. *ovata* batch culture conditions

*Ostreopsis* cf. *ovata* strains CBA 3318 and CBA 3320 were isolated from a bloom that occurred in September 2020 in Passetto, Ancona (Italy) from seawater used for washing macroalgae. Clonal cultures were established, maintained, and grown in 1-L glass bottles containing 400 mL of sterilized F/4 -Si medium at a temperature of 23 ± 1 °C. Light was provided by cool-white, fluorescent bulbs (photon flux of 100 μE m^−2^ s^−1^) with a standard 14:10 light–dark cycle.

### Extraction of *Ostreopsis *cf.* ovata* cell pellets

*Ostreopsis* cf. *ovata* CBA 3318 and CBA 3320 cell pellets (1.0 × 10^6^ total cells) harvested at the exponential phase were extracted according to Ciminiello et al. [26]. In detail, each pellet was added of 3 mL of MeOH:W 1:1 (v/v), vortexed for 30 s then sonicated for 10 min in an ice bath at 20% amplitude (AMP) in pulse mode, and subsequently centrifuged at 6000 rpm for 10 min. The supernatant was decanted and placed in a 15-mL-sized polypropylene centrifuge tube and each pellet was washed twice with 3 mL of the same extracting solvent, sonicated, and centrifuged as described above. The pooled supernatants (9 mL), referred to as the crude extract, were analyzed by LC-HRMS for determining toxin profile and OVTX content. The crude extracts were stored at − 20 °C before being used for spiking experiments.

### Spiking experiments

#### High PLTX/OVTX-a spiking level

Spiking experiments at a relatively high PLTX spiking level (200 ng PLTX/mL) were conducted in duplicate using the following CD polymers: β-CD-HDI, β-CD-EPI, γ-CD-HDI, and γ-CD-EPI or Diaion® HP-20 resin. The experiments were limited to duplicates due to the limited availability of the PLTX standard, which had been discontinued at the time of the study and remains unavailable to date.

1-mL aliquots of filtered toxin-free seawater were added to 20 μl of PLTX standard (200 ng PLTX) or 64 μl of *O.* cf. *ovata* crude extract (about 200 ng OVTX-a) in an Eppendorf tube and then mixed. A 50-mg aliquot of each CD polymer or resin was weighted and added to each Eppendorf tube and incubated for 4 h under agitation on a mixing wheel (450 rpm) at room temperature. Samples were then centrifuged for 10 min at 8000 rpm to remove the seawater, which was decanted. The following solvent mixtures were tested for extracting the CD polymers and resin: MeOH:W 1:1 (v/v) 0.1% AA, MeOH:W 1:1 (v/v) 0.1% FA, MeOH:W 8:2 (v/v), MeOH:W 8:2 (v/v) 0.1% AA, MeOH:W 8:2 (v/v) 8 mM AcNH_4_ buffer pH 9, and MeOH:ACN:W 4:4:2 (v/v/v) 0.1% AA. In details, a 1-mL aliquot of an extracting mixture was added to each Eppendorf tube, and kept for 1.5 h under stirring at 27 °C. Additional extraction times 2, 4 h, and overnight were tested using MeOH:W 8:2 (v/v) 0.1% AA as solvent mixture. Each mixture was centrifuged for 10 min at 8000 rpm and each supernatant was decanted. A second sequential extraction was performed by adding 1 mL of the extracting mixture into the Eppendorf tube containing the CD polymer, vortexed and centrifuged again for 10 min at 8000 rpm; the supernatant resulting from the second extraction was kept separate from the first one. A 5-μL aliquot of each extract was analyzed by LC-HRMS.

#### Low PLTX spiking level

Spiking experiments at a relatively low PLTX spiking level (200 ng PLTX/60 mL, namely 3.3 ng/mL) were conducted in duplicate using γ-CD-HDI and Diaion® HP-20 resin. The concentration was selected based on previous studies reporting *Ostreopsis* cf. *ovata* toxin production mostly ranging from 4 to 70 pg/cell with higher levels up to 238 pg/cell [60] and the Italian Ministry of Health guidelines, which define the Alert phase at *O.* cf. *ovata* cell densities between 10,000–30,000 cells/L up to 100,000 cells/L [40].

60-mL aliquots of filtered toxin-free seawater were added to 200 ng of PLTX standard in 125 mL polypropylene containers and then mixed. 50-mg aliquot of γ-CD-HDI or Diaion® HP-20 resin was added to a container, and it was incubated for 4 h under stirring at 27 °C. The suspension was transferred into a PP tube, then centrifuged at 10,000 rpm for 10 min to remove the seawater. A 1-mL aliquot of MeOH:W 8:2 (v/v) 0.1% AA or MeOH:W: 8:2 (v/v) 8 mM AcNH_4_ buffer pH 9 was added to each PP centrifuge tube and kept under stirring at 27 °C for 2 h and 4 h for Diaion® HP-20 and γ-CD-HDI, respectively, centrifuged for 10 min at 10,000 rpm and the supernatant was decanted. A second sequential extraction was performed also in this case, adding 1 mL of the extracting mixture into the Eppendorf tube containing CD polymers or Diaion® HP-20 resin, vortexed and centrifuged again for 10 min at 10,000 rpm; the supernatant resulting from the second extraction was kept separate from the first one and independently analyzed by LC-HRMS.

Successively, a 1-g aliquot of γ-CD-HDI was added to a container where 200 ng PLTX in 60 mL of filtered toxin-free seawater had been placed. Incubation for 4 h or 120 h under stirring at 27 °C was conducted. The seawater was removed as described above and γ-CD-HDI was extracted with 20 mL MeOH:W 8:2 (v/v) AcNH_4_ 8 mM buffer pH 9 for 4 h, sonicated and centrifuged. The extract was evaporated to dryness using a Heidolph rotary evaporator (Germany), redissolved in 1 mL of 50% aqueous EtOH, filtered through a 0.45-μm filter and analyzed by LC-HRMS.

### Computational study

The computational study was carried out using the PM3 Hamiltonian contained in MOPAC2012 package (http://www.openmopac.net). The starting host geometries were the crystal structures of β-CD and γ-CD obtained from PDB codes 3CGT and 5E70, respectively. The guest geometries consisted of the acyclic end (amide end) of PLTX (C_8’_-C_8_) and the oxolane/dioxabicyclic portion (amine end) located at the other end of the toxin (C_98_-C_115_) (see Fig. 1). Both molecules were manually built and their structures were sequentially minimized at MM2 and PM3 levels without any restrictions using Hyperchem 7.0 software (Hypercube, Inc.).

A series of inclusion geometries with the acyclic end were built by initially placing the C_8’_ atom in the center of the cavity and translating the guest in 0.25 pm steps towards the primary side until the other end (C_8_) reached the center of the cavity, followed by energy minimization of each geometry (PM3). The host was then inverted 180° along the cavity axis and the process was repeated moving the guest towards the secondary side. For the other fragment, only one geometry with the C_115_ atom placed in the center of the cavity was minimized with both orientations of the β-CD and γ-CD hosts. The lowest energy geometries were then visualized using BIOVIA Discovery Studio 2024 (Accelrys).

### Immersion of CD polymers in *Ostreopsis *cf.* ovata* cultures

**Fig. 3:**
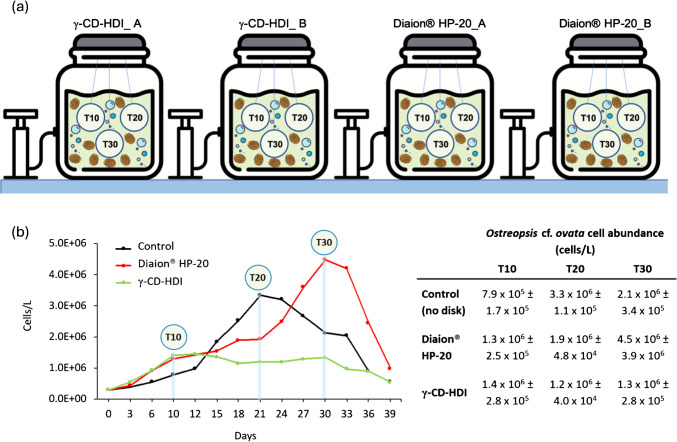
**a** Schematization of different *Ostreopsis* cf. *ovata* microcosm experimental conditions, featuring 3 disks containing passive sampling materials—γ-CD-HDI and Diaion®HP-20—placed in duplicate glass bottles (A and B). T10, T20, and T30 refer to the days of disk collection. **b** Growth curves of *Ostreopsis* cf. *ovata* under different experimental conditions: No passive disk (control – Black line); passive sampling disk containing γ-CD-HDI (green line) and passive sampling disk containing Diaion® HP-20 (red line) with relative table of *O.* cf. *ovata* cell abundance (cells/L) estimated by light microscopy at three sampling times (T10: 10 days; T20: 20 days; T30: 30 days). For each condition, 3 L of 3.0 × 10^5^ cells/L culture (starting inoculum) was established. Mean values and standard errors are shown

Cultures of *O*. cf. *ovata* strain CBA 3320 were established as described above in 5-L-sized glass bottles containing 3 L of 3.0 × 10^5^ cells L^−1^ culture. Cultures were bubbled with air in order to keep cells in suspension. Passive sampling disks containing 1 g of γ-CD-HDI, or 1 g of Diaion®HP-20 were immersed into *O*. cf. *ovata* cultures (3 disks per glass bottle) at day 0, and collected at day 10, 20 and 30 (Fig. 3a). *Ostreopsis* cf. *ovata* cell abundance was estimated at the same sampling time using Sedgewick-Rafter counting chamber under an inverted microscope (ZEISS Axiovert 40CFL) at 400X magnification (Fig. 3b).

The experiment was conducted in 2 replicates due to inherent difficulties associated with cultivating *O.* cf*. ovata* cultures. A culture without passive sampling disks was used as a control to evaluate whether their presence had any effect on the culture growth and toxin production (Fig. 3b black line). Cultures (about 3 L), including culture control, were harvested on day 30 (stationary phase) through vacuum filtration using a 5-μm nylon mesh, or by centrifugation (4000 rpm for 10 min) and cell pellets were extracted as described above. The passive sampling disks were soaked in Milli-Q water for 30 min. Afterwards, the embroidery hoop was opened, and the γ-CD-HDI, or Diaion® HP-20 were transferred into 50mL-sized plastic falcons and stored at − 20 °C. The γ-CD-HDI or Diaion® HP-20 were extracted with 20 mL MeOH:W 8:2 (v/v) AcNH_4_ 8 mM buffer pH 9 for 4 h under stirring at 27 °C, and centrifuged for 10 min at 10,000 rpm, so to obtain the extract. Each extract was evaporated to dryness using a Heidolph rotary evaporator (Germany) and redissolved in 1 mL of 50% aqueous EtOH. A 200-μL aliquot of each extract was filtered through a 0.45-μm filter and analyzed by LC–MS/MS in multiple reaction monitoring (MRM) mode (5 μL injected).

A schematic description of the final optimized conditions to facilitate the practical application of this methodology is reported in Table S1.

### Statistical analyses

Statistical analyses on *O.* cf. *ovata* cell abundances were performed with non-parametric Kruskal–Wallis tests using PAST ver. 4.01, with a *p*-value < 0.05 determining significance [61].

Two-way ANOVA followed by post hoc Tukey HSD tests were conducted on LC-HRMS data to evaluate PLTX recovery across different extraction mixtures and incubation times, as well as OVTXs recovery using different CD polymers. All analyses were conducted using Python and executed via Google Colab, with a *p*-value < 0.05 considered significant.

### Liquid chromatography–high resolution mass spectrometry (LC-HRMS)

LC-HRMS analyses were carried out on a hybrid linear ion trap LTQ Orbitrap XL™ (SN 01719B) Fourier Transform Mass Spectrometer (FTMS) equipped with an ESI ION MAX™ source coupled with a Dionex Ultimate 3000 quaternary HPLC system (Thermo-Fisher, San Jose, CA, USA). LC separation was achieved on a Kinetex 2.6 μm, 100 Å, 100 × 2.1 mm (Phenomenex, Torrance, CA, USA) HPLC column, eluted at a flow rate of 0.2 mL/min at room temperature with water (A) and ACN:W 95:5 (v/v) (B) both containing 30 mM AA. The following gradient was used: *t* = 0 min, 20% B; *t* = 10 min, 100%B; *t* = 15 min, 100%B; *t* = 16 min, 20%B; *t* = 17 min, 20%B; re-equilibration time was 10 min. Source parameters were spray voltage 4.8 kV, capillary temperature 360 °C, capillary voltage 36 V, sheath gas 60 and auxiliary gas 21 (arbitrary units), and tube lens voltage 100 V. Based on the previously observed ionization behavior of PLTX and OVTXs, which is characterized by the formation of prominent [M + H + Ca]^3+^ ions for each analogue (Fig. S2, [62]), HR full-scan experiments were acquired in the *m*/*z* range 800–1500 at resolving power (RP) 60,000 (FWHM at *m*/*z* 400). Extracted ion chromatograms (XICs) were obtained by selecting the exact mass of the mono-isotopic peak of the [M + H + Ca]^3+^ ion cluster of each toxin, namely OVTX-a at *m/z* 895.8195; OVTX-b at *m/z* 910.4949; OVTX-c at *m/z* 915.8266; OVTX-d/e at *m/z* 901.1511; and isobaric PLTX at *m/z* 906.4828. Elemental formulae were assigned with Thermo Xcalibur software v2.2 SP1.48 (Thermo Fisher, San José, CA, USA) within a mass tolerance of 5 ppm. In the absence of relevant standards, OVTX quantitation was accomplished by using the PLTX calibration curve and assuming the same molar response for PLTX and OVTXs. Toxin recovery percentage (%) was calculated by the following formula:

$$\text{recovery percentage }\left(\%\text{}\right)= [(\text{observed concentration})/(\text{spiked concentration})] \times 100$$

For each set of experiments, a seven-level calibration curve of PLTX standard in 50% aqueous EtOH (1000, 500, 250, 125, 62.5, 31.2, 15.6 ng/mL) was prepared and used for quantitation (*R*^2^ = 0.999). The limit of detection (LOD) and quantitation (LOQ) were 2 and 12 ng/mL, respectively.

### Liquid chromatography–tandem mass spectrometry (LC–MS/MS)

LC–MS/MS experiments were conducted on a Triple quadrupole MS spectrometer Agilent 6470 (Santa Clara, CA, USA) equipped with an Agilent Jet Stream Source. The same column, mobile phases, and gradient elution described in the “Immersion of CD polymers in *Ostreopsis* cf. *ovata* cultures” section were used. Source parameters were gas temperature 350 °C, gas flow 13 L/min, nebulizer pressure 20 psi, sheath gas temperature 350 °C, sheath gas flow 11 L/min, capillary voltage 5000 V, and nozzle voltage 2000 V. MRM experiments were acquired using the transitions *m*/*z* 906.8 > 327.1, 906.8 > 1169.1 for PLTX and *m*/*z* 896.1 > 327.1, and 896.1 > 1153.1 for OVTX-a. LOQ was 40 ng/mL.

## Results and discussion

In the present study, the effectiveness of CD polymers in capturing PLTX-like compounds from seawater was evaluated by examining several experimental parameters, including solvent mixture composition, extraction time, recovery of OVTX analogues, the effect of pH on the extracting solvent, and toxin concentration. Throughout all experiments, Diaion® HP-20 resin was used as a control. This resin is widely recognized for its efficiency in adsorbing various lipophilic marine toxins, such as okadaic acid, pectenotoxins, ciguatoxins, cyclic imines, yessotoxins, and azaspiracids with reported recovery rates ranging from 36 to 80% [41–43, 63–73]. Additionally, Diaion® HP-20 resin has been tested in passive sampling hydrophilic toxins such as domoic acid, saxitoxins, and anatoxins [43, 65, 74], showing moderate recovery (~ 45%). Although no positive data are currently available regarding its performance in the monitoring of PLTX-like compounds [68], its broad-spectrum adsorption capacity makes it a well-established and cost-effective benchmark [65].

### Spiking experiments

The experimental design was focused on testing the ability of different insoluble CD polymers (β-CD-HDI, β-CD-EPI, γ-CD-HDI, γ-CD-EPI) versus the conventional Diaion® HP-20, to capture PLTX-like compounds from seawater at the selected concentration levels 200 and 3.3 ng/mL (corresponding to the OVTX content estimated during the *O*. cf. *ovata* alert phase [40]) by analyzing only the toxin extracted from CD polymers or Diaion® HP-20 resin and thus bypassing the labour-intensive process of handling large seawater volumes. Toxin recoveries were evaluated after the extraction of CD polymers testing different extracting solvent mixtures and different extraction times.

#### Effect of the extracting solvent mixture

Different binary and ternary solvent mixtures with or without AA were tested for the extraction of CD polymers after incubation in PLTX-spiked seawater (200 ng/mL). The selection of the solvent mixture was guided by data reported by Tartaglione et al. [44] indicating that MeOH:W 8:2 0.1% TFA was the preferred system to elute PLTX from HLB SPE cartridges. However, TFA was substituted with AA based on the strong degradation effect of TFA on PLTX observed by Mazzeo et al. [75].

**Fig. 4:**
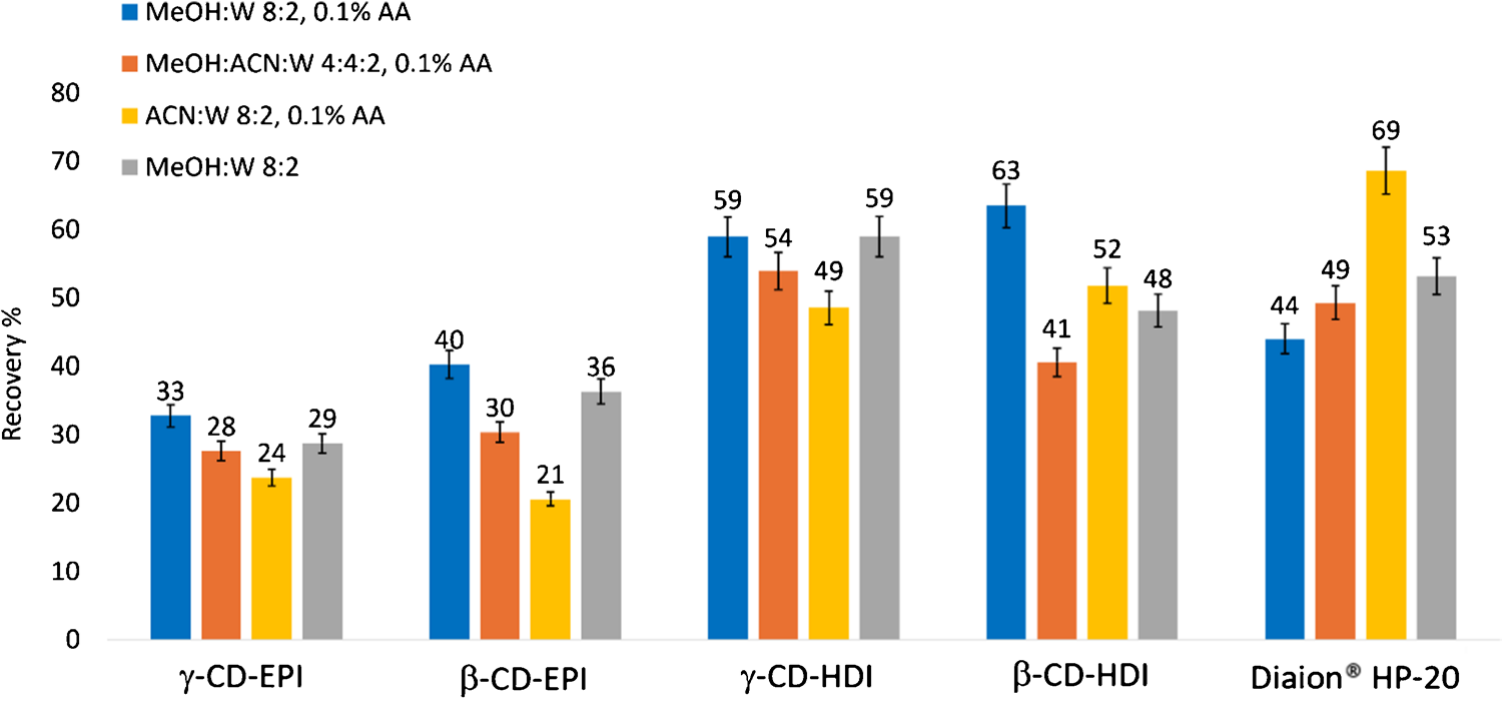
Recovery percentage of PLTX captured by CD polymers or Diaion® HP-20 resin and extracted using different solvent mixtures in the frame of spiking experiments. PLTX spiking level was 200 ng/mL and extraction time was 1.5 h. Each bar corresponds to the mean of two technical replicates. Each bar corresponds to the mean of two technical replicates with an instrumental variability of response equal to 5%

All the available CD polymers were screened to assess the influence of the cavity size, related to the number of glucose units (7 in β-CD and 8 in γ-CD), as well as the polarity of the spacer arms in the polymer structure, on their capture ability. In general, HDI-based CD polymers showed higher toxin recoveries (41–63%) than the EPI counterparts (21–40%) with the Diaion® HP-20 resin control showing intermediate recovery rates (44–53%) (Fig. 4). To validate these findings, the two-way ANOVA followed by post hoc Tukey HSD test was performed. Results revealed that the sorbent material (*p* < 0.001) and the interaction between the extraction mixture and sorbent material (*p* < 0.001) significantly affected the PLTX recovery. In particular, the post hoc comparisons using the Tukey HSD test confirmed that β-CD-HDI achieved significantly higher recovery than γ-CD-EPI (*p* = 0.0001) and β-CD-EPI (*p* = 0.0005), with no significant difference from Diaion® HP-20 resin. Similarly, γ-CD-HDI significantly outperformed γ-CD-EPI (*p* = 0.0021) and β-CD-EPI (*p* = 0.0076), also showing no significant difference from Diaion® HP-20 resin.

**Fig. 5:**
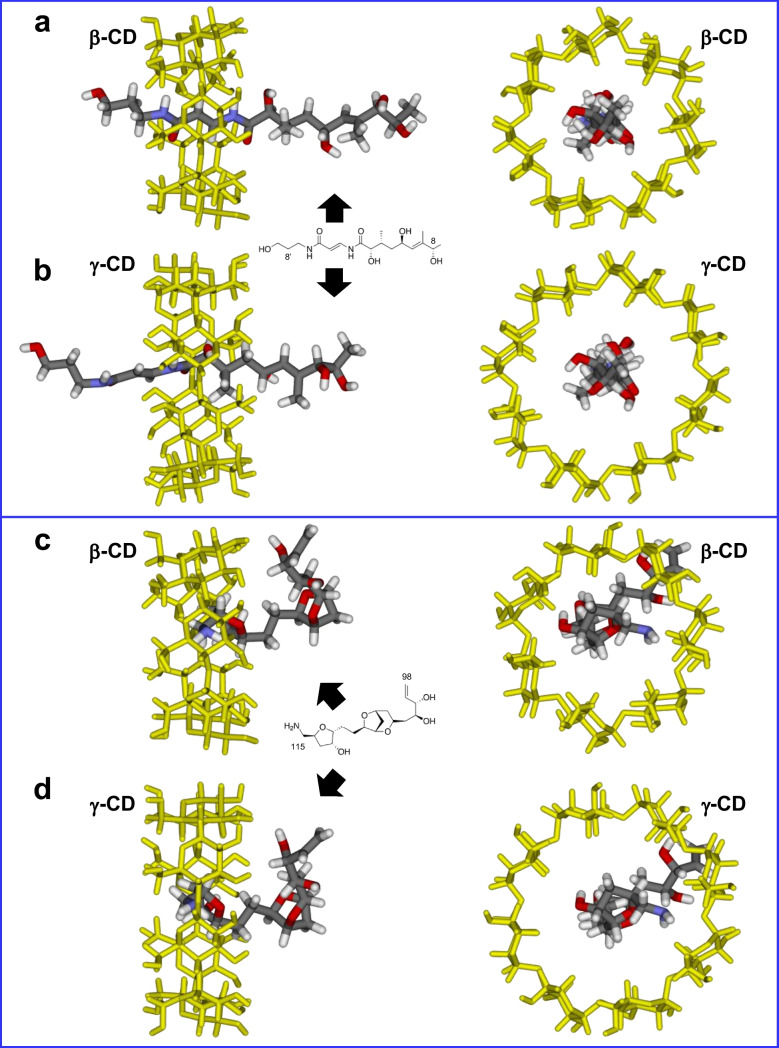
Lowest energy geometries (PM3) of the inclusion complexes between β-CD and γ-CD with both ends of the PLTX molecule: **a**, **b** C_8’_-C_8_ acyclic part, **c**, **d** oxolane/dioxabicyclic region at the opposite end (see Fig. 1 for the complete PLTX structure and Section "Computational study" for details). Left: lateral view. Right: Top view. The cyclodextrin (CD) hosts have been highlighted in yellow

In insoluble CD-based polymers, adsorption takes place either through the formation of intracavity inclusion complexes or by interaction with extra cavity binding sites formed by the spacer network, provided they are accessible [76, 77]. Intracavity complexation is influenced by the cavity size while interactions with extra cavity sites are governed by the degree of cross-linking and chemical functionalities in the structure of the spacers. In the capture of PLTX by the studied CD polymers, a possible explanation for the results presented in Fig. 4 could lie in the higher affinity of PLTX for the HDI spacers within the polymer backbone (which contains six methylene groups connected to CD by -O(C=O)NH- groups) (Fig. 2). The isocyanate and hydroxyl groups of CD-HDI polymers promote hydrogen bonding interactions with the hydroxyl functionalities of the toxin, and therefore, the nature of the spacer arms certainly plays a decisive role in capturing PLTX as has been observed in other CD-based polyurethanes [78]. To study the influence of the intracavity complexation, we performed theoretical calculations of the interaction of both ends of the linear PLTX backbone with unsubstituted β-CD and γ-CD. Semi-empirical methods have the advantage of being relatively low computationally demanding and, as such, are a widely used tool to predict the possible structures of CD inclusion complexes [79, 80] since they can provide helpful insights on complex formation and conformation stabilities of complex guests such as cyclic peptide cyanotoxins [81]. Our results suggest that the host/guest interactions occur predominantly with the acyclic end of PLTX (along the C_8’_-C_8_ part structure, Fig. 1), which penetrates deeper in the γ-CD cavity than in β-CD (Fig. 5a, b). In contrast, the bulkier oxolane/dioxabicyclic portion located at the other end of the toxin only interacts very weakly with the external hydroxyl rims of the CD molecule (Fig. 5c, d).

Based on these theoretical observations, γ-CD-HDI, with its wider internal cavity, could be expected to perform better than β-CD-HDI in capturing PLTX. However, experimental evidence showed no significant difference in PLTX recovery percentages measured for β-CD-HDI (41–63%) and γ-CD-HDI (49–59%) after the extraction process (Fig. 4). Additionally, since host/guest interactions with linear polar molecules are generally weak [82], inclusion complexation appears to play less influence in the toxin recoveries than the nature of the spacer arms linking the CD molecules in the polymer.

The extracting solvent mixture MeOH:W 8:2 (v/v) 0.1% AA provided the highest PLTX recoveries with all tested CD polymers, whereas the less polar mixture ACN:W 8:2 (v/v) 0.1% AA performed better when using Diaion® HP-20. This could reflect the nature of the interactions taking place between PLTX and the CD polymers since more polar solvents are needed to break the hydrogen bonding interactions as compared to Diaion® HP-20, where hydrophobic interactions with the polystyrene backbone are predominant.

#### Effect of the extraction time

**Fig. 6:**
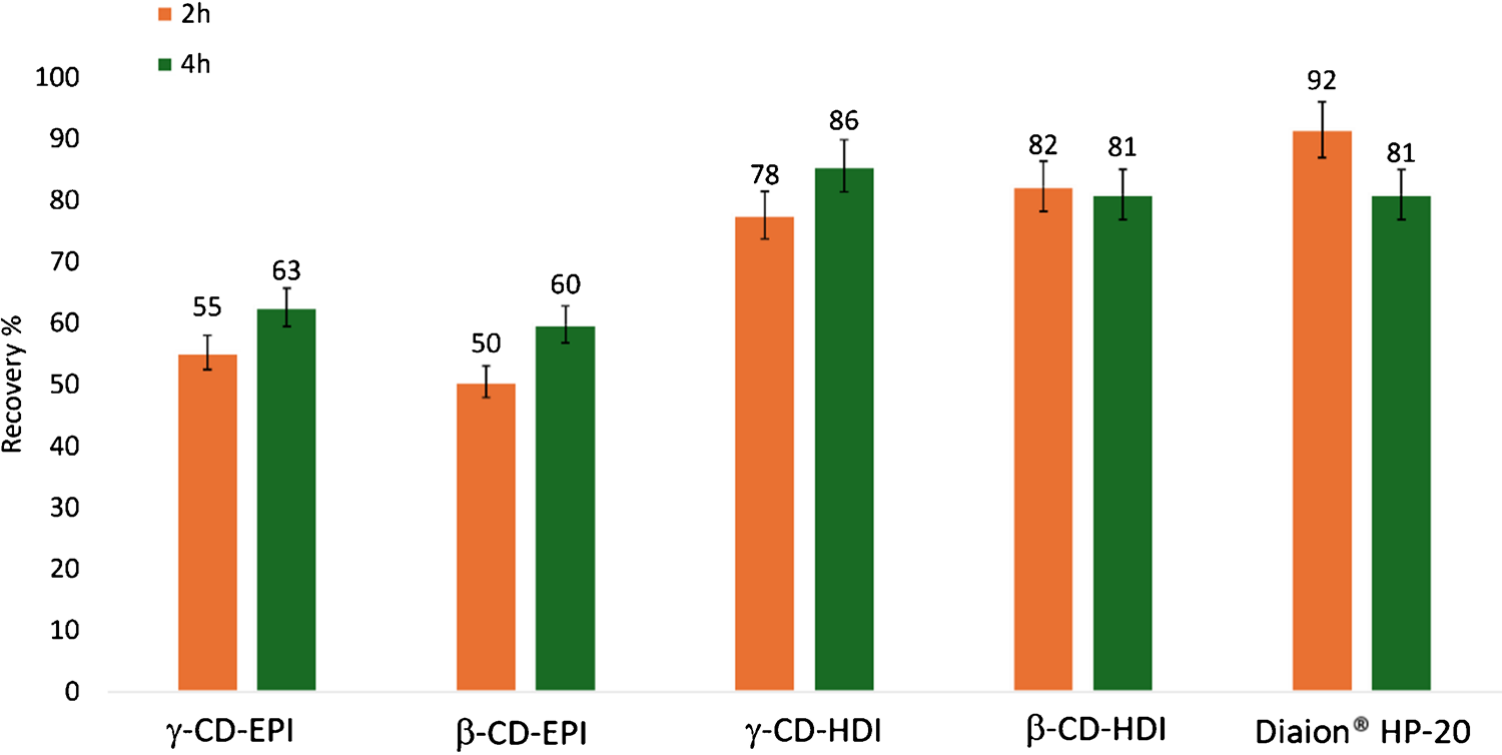
PLTX recoveries obtained with the different CD polymers and Diaion® HP-20 resin at different extraction times tested (2 h and 4 h). PLTX spiking level was 200 ng/mL and MeOH:W 8:2 (v/v) 0.1% AA was the extraction mixture. Each bar corresponds to the mean of two technical replicates with an instrumental variability of response equal to 5%

To assess the impact of extraction time on PLTX recovery from each CD polymer, the most effective solvent mixture (MeOH:W 8:2 (v/v) with 0.1% AA) was used extending the extraction time to 2 and 4 h (Fig. 6). Interestingly, only γ-CD-HDI showed a slight, yet not statistically significant (*p* > 0.05), increase in toxin recovery with extended extraction times (Fig. 6), improving from 78% (2 h) to 86% (4 h). Therefore, γ-CD-HDI (versus Diaion® HP-20) was chosen to evaluate whether further increasing the extraction time, specifically an overnight extraction, would improve the recovery. However, no appreciable improvement in toxin recovery was observed (Fig. S3). The whole of these results suggested that extraction times of 2 and 4 h with MeOH:W 8:2 (v/v) 0.1% AA were the best conditions for extracting PLTX from Diaion® HP-20 and all CD polymers, respectively.

#### Testing CD polymers for OVTX analogues’ recovery

**Fig. 7:**
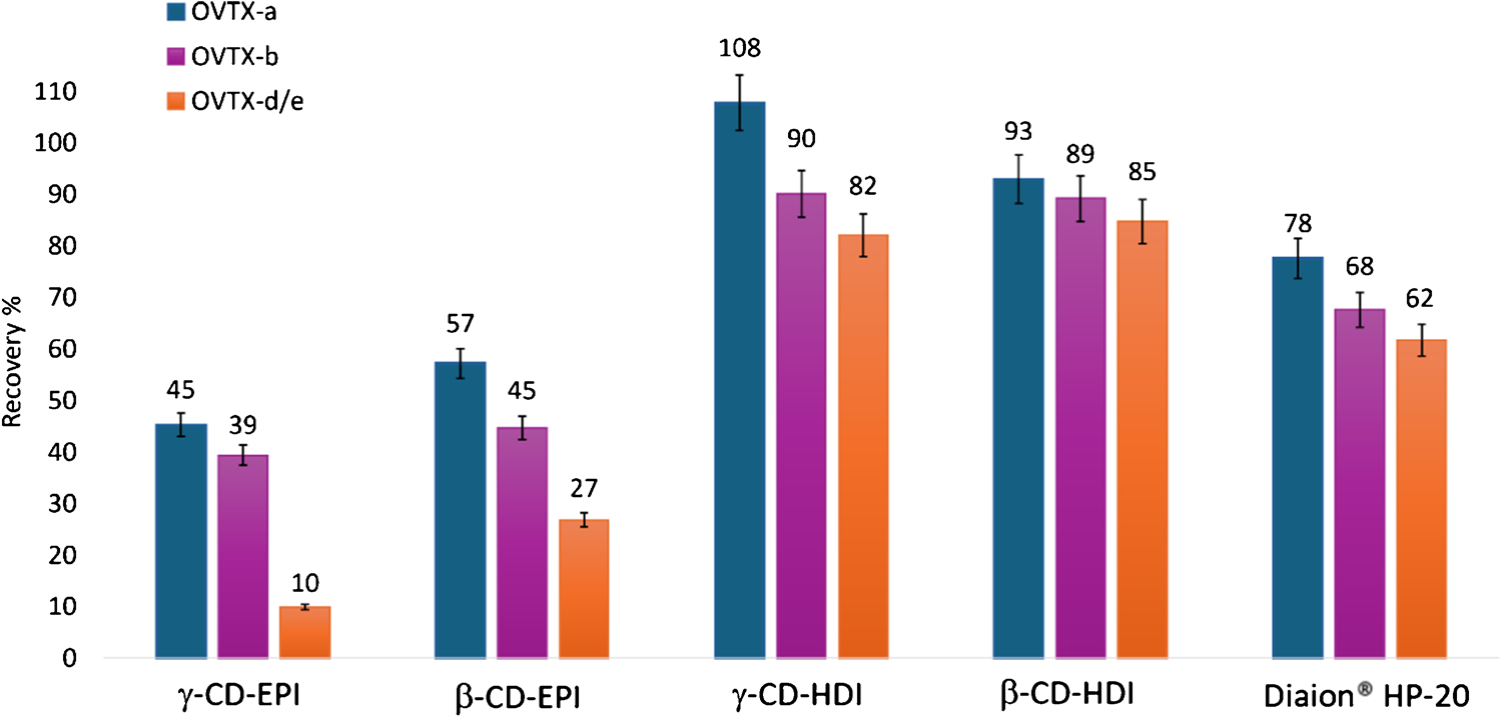
Recovery percentage of OVTX-a, -b, and -d/-e obtained testing different CD polymers or Diaion® HP-20 resin in the frame of spiking experiments with an *O.* cf. *ovata* extract. OVTX-a spiking level was 200 ng/mL and extraction time was 1.5 h. Each bar corresponds to the mean of two technical replicates with an instrumental variability of response equal to 5%

To assess whether the CD polymers previously tested for PLTX capture could also bind its structural analogues, OVTXs (Fig. 1), spiking experiments (200 ng/mL) were conducted using *O.* cf. *ovata* extract (strain CBA 3318) due to the unavailability of a commercial OVTXs standard. This strain, originally collected from Ancona, Italy, in 2020, exhibited the most commonly reported toxin profile in the Mediterranean region. OVTX-a was the predominant component, accounting for 55% of the total toxin abundance, alongside OVTX-b to -e and isobaric PLTX (Table S2). Overall toxin content of strain CBA3318 was 51.3 pg OVTXs/cell (28.3 pg OVTX-a/cell, Table S2). Consistently with the results obtained for PLTX, LC-HRMS analyses and revealed that CD-HDI polymers allowed to achieve higher recoveries (82–108%) than CD-EPI (10–57%), with γ-CD-HDI (the one featuring the wider ring of glucose subunits) providing slightly higher, recoveries than β-CD-HDI (Fig. 7). The two-way ANOVA confirmed that both sorbent material type (*p* < 0.001) and OVTX analogue (*p* < 0.001) significantly and independently affect recovery. Subsequent post hoc Tukey HSD tests showed that β-CD-HDI and γ-CD-HDI exhibited significantly higher recoveries than γ-CD-EPI and β-CD-EPI (*p* < 0.0001 for all comparisons). Notably, while some differences between CD-HDI polymers and Diaion® HP-20 control are visually appreciable, only the difference of γ-CD-HDI from the control was statistically significant (*p* = 0.0243) (Fig. 7). The use of MeOH:W 8:2 (v/v) 0.1% AA as extracting solvent again showed promising in achieving high recoveries (82–108%). Interestingly, the recovery efficiency varied slightly (*p* = 0.1) among the OVTX analogues (Fig. 7) following a trend where OVTX-a exhibited the highest recovery, followed by OVTX-b and then OVTX-d/-e. These results strengthen the initial observations and indicate that γ-CD-HDI remains a promising candidate for efficient toxin capture.

The ability to differentiate between OVTX analogues highlighted the importance of selecting appropriate CD polymers for toxin capture and the potential for tailored isolation methods for different toxin variants. It is worth noting that recovery rates measured for OVTXs are generally higher than those measured for PLTX standard (Figs. 4 and 7). This difference may be attributed to the higher methanol content used in the spiking experiments with the *O*. cf. *ovata* crude extract (3.2% vs. 1.0% for the PLTX standard), which could have enhanced toxin solubility in the aqueous solution and, consequently, influenced recovery rates.

#### Effect of the pH of the extracting solvent mixture

**Fig. 8:**
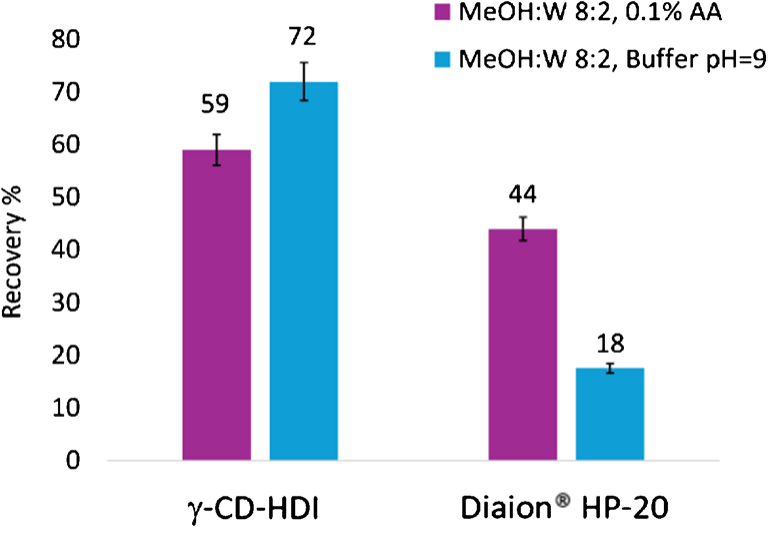
Recovery percentage of PLTX obtained in acidic and alkaline conditions (MeOH:W 8:2 (v/v) 0.1% AA, MeOH:W 8:2 (v/v) 8 mM AcNH_4_ buffer pH 9 using γ-CD-HDI and Diaion® HP-20 resin. PLTX spiking level was 200 ng/mL and extraction time was 1.5 h. Each bar corresponds to the mean of two technical replicates with an instrumental variability of response equal to 5%

A further investigation was carried out to compare the performance of the most promising CD polymer, γ-CD-HDI, versus Diaion® HP-20 using an alkaline extracting mixture (MeOH:W 8:2 (v/v) 8 mM AcNH_4_ buffer, pH 9). The alkaline extracting mixture provided a higher PLTX recovery percentage compared to the acidic mixture previously used (72% versus 59%, respectively) (Fig. 8). This indicated that the pH of the extracting solvent can influence toxin recovery, with alkaline conditions showing slightly better performance. The two-way ANOVA confirmed that the type of material used significantly affected toxin recovery (*p* = 0.0031) and that the efficiency of the extraction from each material was significantly influenced (*p* = 0.0230) by the pH of the solvent mixture. In particular, the post hoc comparisons using the Tukey HSD test confirmed that γ-CD-HDI provided significantly higher recovery than Diaion® HP-20 under alkaline conditions (*p* = 0.0113), while no significant difference was observed under acidic conditions (*p* = 0.1525). Furthermore, Diaion® HP-20 recovery decreased significantly under alkaline conditions than under acidic conditions (*p* = 0.0424). Additionally, under the tested conditions, results confirmed again that γ-CD-HDI continued to be a promising candidate for toxin capture, potentially outperforming Diaion® HP-20.

#### Effect of the toxin concentration

Spiking experiments also aimed to explore how variations in PLTX concentration in seawater influenced toxin recovery from the selected γ-CD-HDI versus Diaion® HP-20 after extraction in acidic or alkaline conditions. In all cases, at a low spiking level of 200 ng PLTX in 60 mL seawater (3.3 ng PLTX/mL), PLTX recovery percentages were very low, with alkaline conditions yielding a slightly higher recovery (10% for γ-CD-HDI versus 13% for Diaion® HP-20) compared to acidic conditions (2% for γ-CD-HDI versus 3% for Diaion® HP-20).

A possible influence of the amount of sorbent material used in the experiments was also considered. Specifically, 1 g of γ-CD-HDI (instead of 50 mg), 4 h incubation time and alkaline conditions for the extraction (Table S3) were tested. PLTX recovery increased from 10 to 29%, suggesting that the amount of γ-CD-HDI affected the recovery efficiency. Then, the potential influence of incubation time was also explored by increasing the incubation time to 120 h (Table S3). PLTX recoveries with extended incubation time turned out to be 26%. This suggested that extended incubation times do not improve recovery rates appreciably.

### Deployment of cyclodextrin polymers in *Ostreopsis *cf.* ovata* cultures

The next step towards bridging the gap between spiking laboratory experiments and biological complexity of natural systems involved setting up tightly controlled microcosms of *O.* cf. *ovata* designed to mimic natural ecosystem conditions. In this experiment, disks of γ-CD-HDI and Diaion® HP-20 were immersed in *O.* cf. *ovata* cultures (~ 3-L glass bottles) (Fig. 3). The purpose of this experiment was to provide insights on the efficiency of passive sampling CD materials in capturing OVTXs released by the producing organism in the growth medium where a variety of other metabolites are present and possibly competing for binding.

OVTXs captured by γ-CD-HDI and Diaion® HP-20 in microcosms at days 10, 20, and 30 were measured by LC–MS/MS (Table 1) along with the OVTX content in cell pellets at harvest, day 30 (Table S4). Toxin captured by γ-CD-HDI and Diaion® HP-20 (Table 1) were expressed both as OVTX-a concentration per culture volume (ng/L) and on a per cell basis (pg/cell). Interestingly, OVTXs were not detected in any of the polymers’ extracts on day 10. This suggested that toxin release in the first stage of *O*. cf. *ovata* growth was scarce under the experimental conditions used. Alternative explanations of this finding include the low intrinsic solubility of ovatoxins in seawater, which may favor their retention within algal cells, or the affinity of ovatoxins for adsorption onto suspended particulate matter, which could lead to their rapid removal from the water column [27].

**Table 1:** OVTX-a captured by the passive sampling disks immersed in *O.* cf. *ovata* cultures and collected on day 10, day 20 and day 30 extracted and analyzed by LC–MS/MS. Each experiment was conducted in duplicate (A and B). (n.d. = not detected)

Samples	OVTX-a γ-CD-HDI		OVTX-a Diaion®HP-20	
	ng/L	pg/cell	ng/L	pg/cell
A_day10	n.d	n.d	n.d	n.d
B_day10	n.d	n.d	n.d	n.d
A_day20	97.9	0.08	25.6	0.01
B_day20	119.4	0.10	65.0	0.03
A_day30	240.9	0.15	61.8	0.01
B_day30	401.9	0.38	66.6	0.12

On the contrary, OVTX-a was detected in γ-CD-HDI and Diaion® HP-20 extracts on days 20 and 30. In both cases, toxin contents exhibited an increasing trend over time. Specifically, OVTX-a levels recovered by γ-CD-HDI increased from 98 to 119 ng/L (or 0.08–0.10 pg/cell) at day 20 to 241 to 402 ng/L (or 0.15–0.38 pg/cell) at day 30 (Table 1). One possible explanation of these results could be the toxin percentage released in the medium during the growth. As suggested by Guerrini et al. [83] and Brissard et al. [34], toxins released by *O*. cf. *ovata* in the growth medium increase as the culture day progresses, being the highest at the end of the stationary phase [84, 85]. A further possible aspect to consider is that, during the aging process, *O*. cf. *ovata* cells may enter a state of cellular senescence leading to cell breakage and subsequent release of toxins into the medium.

The comparison between the toxin content of *O*. cf. *ovata* cell pellet collected at the end of microcosm experiments (Table S4) in the control culture grown in the absence of polymers (3.0 pg/cell) with those measured in the presence of γ-CD-HDI and Diaion® HP-20 disks revealed interesting insights regarding the effect of sorbent materials on toxin production. When *O*. cf. *ovata* was grown in the presence of γ-CD-HDI disks, the toxin production increased to 12.2 pg/cell (mean of two replicates, Table S4). This suggests that the presence of CD polymer disks somehow stimulated and enhanced toxin production of *O*. cf. *ovata* compared to the control culture. Instead, for the cultures grown in presence of Diaion® HP-20, no clear trend could be deduced since biological replicates provided toxin content spreading over a quite large range (Table S4).

*O.* cf. *ovata* cell abundance estimated at different sampling times (Fig. 3b) showed no significant differences (Hc = 1.8; *p* = 0.9) indicating that the disks, containing different passive sampling materials, had no effect on cell growth.

## Conclusions

The whole of these results highlights that various factors, including toxin concentration, sorbent material amount, and extraction time, play an important role in the efficiency of toxin recovery from CD-based sorbent materials. Spiking experiments conducted at high concentrations of PLTX or OVTXs (200 ng/mL) demonstrated that CD polymers, effectively trapped PLTX-like toxins, with good recovery yields after extraction. In contrast, experiments at lower PLTX concentration (3.3 ng/mL) resulted in considerably reduced recovery percentages.

Alkaline conditions generally appeared to provide a higher recovery than acidic conditions and increasing the amount of sorbent material and incubation time during extraction also improved recovery rates. Particularly, for γ-CD-HDI polymer, PLTX recovery at the lowest spiking level (3.3 ng toxin/mL) was 10% under alkaline conditions versus 2% in acidic conditions, whereas at the highest spiking level (200 ng toxin/mL) PLTX recovery reached 72% under alkaline extraction conditions, compared to 59% under acidic conditions. The lowest measured concentration of toxin passively collected was 20.1 ng in 1 mL MeOH:W 8:2 8 mM AcNH_4_ buffer pH 9 extract (mean value of two replicates). This value, corresponding to the 10% recovery from a 200 ng toxin spike in 60 mL seawater (Table S3), exceeded the instrumental LOQ (12 ng/mL) indicating the suitability of the LC-HRMS method in the detection of PLTXs at such level.

These findings support the potential of CD polymers for passive sampling of PLTX-like compounds at high concentrations. However, further optimization may be required to improve recovery rates for samples with lower toxin concentrations.

National guidelines, established by the Italian Ministry of Health in coordination with other public authorities, indicate thresholds based on the abundance *O*. cf. *ovata* cells per liter of seawater to assess and manage the risk associated with blooms along Italian coastlines. These guidelines delineate three phases—routine, alert, and emergency—corresponding to cell concentration thresholds of ≤ 10,000 cells/L, 10,000–30,000 cells/L, and > 100,000 cells/L, respectively [40]. In microcosm experiments, OVTXs were captured by γ-CD-HDI at levels associated with *O*. cf. *ovata* growth, with the first signals being detected on day 20 when the abundance was approximately 1.0 × 10^6^ cells in each 3-L glass bottles (corresponding to 300,000 cells/L).

Our findings suggest that γ-CD-HDI polymers may effectively capture OVTXs during intense *O.* cf. *ovata* blooms. However, to date, they do not meet the requirements of environmental monitoring demands for early warning systems for *O*. cf. *ovata* blooms. In fact, despite the lack of direct detection of OVTXs in the first phase of cells growth, most of the intoxication events associated with *O.* cf *ovata* have been documented in the transition from the exponential growth to the stationary phase of the bloom [24, 25]. This suggests that ovatoxins may still become bioavailable under certain conditions, potentially through mechanisms such as aerosolization [13, 27] or other environmental pathways.

It is important to note that a low concentration of *Ostreopsis* spp. cells does not necessarily lead to the development of a bloom in the sea. Therefore, while CD polymers could be valuable tools for direct toxin capture during bloom events—especially when highly toxin-producing strains are present—the implementation of additional monitoring strategies, as well as the planning of other activities tailored to different bloom phases, is crucial to effectively manage the risks associated with *O.* cf. *ovata* blooms and to support the health surveillance system.

## Supplementary Information

Below is the link to the electronic supplementary material.

ESM 1 (DOCX 669 KB)

## Data Availability

Should any raw data files be needed in another format they are available from the corresponding author upon reasonable request.
