# Supplementary material for: Evaluation of the efficiency of cyclodextrin polymers as sustainable sampling material for catching palytoxin-like compounds in seawater
Source: Mikrochim Acta. 2025 Sep 10;192(10):653. doi: 10.1007/s00604-025-07507-0 (PMC12423253; doi:10.1007/s00604-025-07507-0)
Supplement: Supplementary file 1 — (DOCX 669 KB) [file 604_2025_7507_MOESM1_ESM.docx]

# Supporting Information

**Evaluation of the efficiency of cyclodextrin polymers as sustainable sampling material for catching palytoxin-like compounds in seawater**

Antonella Miglione^a,1^**,** Chiara Melchiorre^a,b,1^, Samuela Capellacci^c,1^, Luciana Tartaglione^a,^*, Michela Varra^a^, Alex Fragoso^d^, Silvia Casabianca^c^, Mabel Torréns^d^, Jorge Diogène^e^, Antonella Penna^c^, Carmela Dell’Aversano^a,b^, Mònica Campàs^e^

^a^ Department of Pharmacy, University of Naples Federico II, School of Medicine and Surgery, Via D. Montesano 49, 80131 Naples, Italy

^b^ NBFC, National Biodiversity Future Center, Palermo 90133, Italy

^c^ Department of Biomolecular Sciences, University of Urbino, Campus E. Mattei, Urbino, 61029, Italy

^d^ Departament d’Enginyeria Química, Universitat Rovira i Virgili, Av. Països Catalans 26, 43007, Tarragona, Spain

^e^ Marine and Continental Waters, IRTA, Ctra. Poble Nou km 5.5, 43540, La Ràpita, Spain

^1^Antonella Miglione, Chiara Melchiorre and Samuela Capellacci contributed equally to this study.

* Corresponding author: Luciana Tartaglione. Tel.: +39 081-678133

E-mail address: [luciana.tartaglione@unina.it](mailto:luciana.tartaglione@unina.it)

# Tables and Figures:

**Table S1** Schematic overview and summary of the experimental conditions, highlighting key steps and practical recommendations for the effective implementation of the methodology presented in Sections 2.7 and 3.2.

| **Step-by-step workflow** | |
| --- | --- |
| Passive Sampling Disks (Disk) preparation | Place a 1 g of γ-CD-HDI bulk (or Diaion® HP-20) between two layers of 1-μm nylon mesh secured between two cylindrical PVC rings (4 cm diameter) (Fig. S1) |
| Disk activation | Activate the disk by soaking it in MeOH for 15 min, then rinse with Milli-Q water |
| Disk deployment | Immerse the disk in *Ostreopsis cf. ovata* culture* (3 disks per glass bottle) on day 0 (Fig. S1) |
| Disk collection (on T10, T20, and T30) ** | After exposure, soak the disk in Milli-Q water for 30 min. Then, open the embroidery hoop and transfer the γ-CD-HDI, (or Diaion® HP-20) material into 50 mL plastic centrifuge tubes |
| CD polymer (or Diaion® HP-20) extraction | Add 20 mL MeOH:W 8:2 (v/v) 8 mM AcNH_4_ buffer (pH 9) to the tube containing the γ-CD-HDI (or Diaion® HP-20). Stir at 27 °C for 4 h, and centrifuge for 10 min at 10000 rpm to obtain the extract. Each extract was evaporated to dryness using a rotary evaporator and redissolved in 1 mL of 50% aqueous EtOH |
| LC-MS analyses | Filter a 200-μL aliquot of the extract through a 0.45-μm filter and analyze using LC-MS/MS in multiple reaction monitoring (MRM) mode as described in section 2.10 |

* Starting inoculum = 3.0 x 10^5^ cells/L culture. A continuous aeration of *O.* cf*. ovata* cultures is required to maintain cells in suspension

** T10, T20 and T30 refer to the days of disk collection (T10: 10 days; T20: 20 days; T30: 30 days)

**Table S2** Relative abundance of ovatoxins (OVTXs) and isobaric palytoxin (isob PLTX) in the toxin profile of *O*. cf. *ovata* strain CBA 3318

| 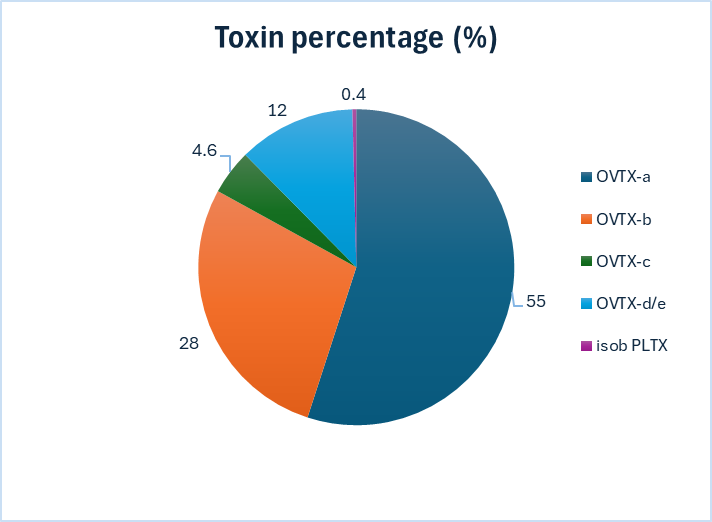 | | |
| --- | --- | --- |
| OVTXs | **pg/cell** | **Percentage (%)** |
| OVTX-a | 28.3 | 55 |
| OVTX-b | 14.2 | 28 |
| OVTX-c | 2.4 | 4.6 |
| OVTX-d/e | 6.2 | 12 |
| isob PLTX | 0.2 | 0.4 |

**Table S3** PLTX recoveries obtained in low spiking level experiments (3.3 ng PLTX/mL) using different amounts of γ-CD-HDI (50 mg or 1 g), different incubation time (4 h or 120 h) and extracting solvent mixtures MeOH:W 8:2 8mM AcNH_4_ buffer pH 9. Each recovery % corresponds to the mean of two technical replicates

| **Incubation time** | **γ-CD-HDI amount** | **PLTX recovery (%)** |
| --- | --- | --- |
|  |  | **MeOH:W 8:2**  **8mM AcNH_4_ buffer pH 9** |
| 4h | 50 mg | 10 |
|  | 1 g | 29 |
| 120h | 1 g | 26 |

**Table S4** OVTX-a content in *O*. cf. *ovata* cell pellets (strain CBA 3320) collected at the end of mesocosm experiments (day 30), extracted and analyzed by LC-MS/MS. Ctrl = *O*. cf. *ovata* culture control, grown without passive sampling disks

|  | ***O*. cf. *ovata* cells** | **pg/cell** |
| --- | --- | --- |
| γ-CD-HDI_A | 3930000 | 11.3 |
| γ-CD-HDI_B | 2510000 | 13.0 |
| Diaion®HP-20_A | 21500000 | 2.9 |
| Diaion®HP-20_B | 1260000 | 8.2 |
| Ctrl | 6150000 | 3.0 |


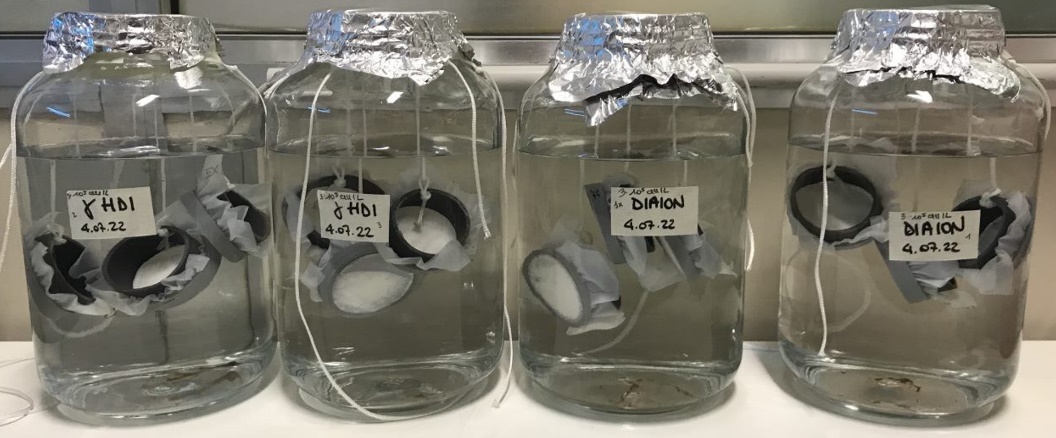


**a)**

**b)**

**
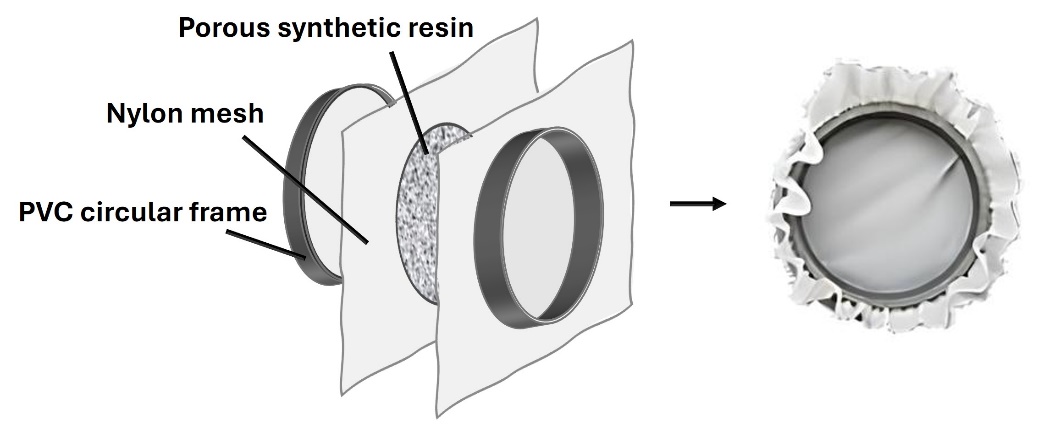
**

**Fig. S1** Passive sampling devices. a) Passive sampling disk assembly; b) Disks exposed to *Ostreopsis* cf. *ovata* CBA 3320 cells. Polyvinyl chloride (PVC)

**Fig. S2** HR full MS spectrum of OVTX-a acquired in the range *m*/*z* 800–1500 on a hybrid linear ion trap LTQ Orbitrap XL™ (SN 01719B) Fourier Transform Mass Spectrometer (FTMS) (See Experimental section 2.9)

γ-CD-HDI

**Fig. S3** Recoveries percentage of PLTX obtained with γ-CD-HDI and Diaion® HP-20 resin at 4 h and overnight extraction times. PLTX spiking level was 200 ng/mL of seawater and MeOH:W 8:2 (v/v) 0.1% AA was the extraction mixture. Each bar corresponds to the mean of two technical replicates with an instrumental variability of response equal to 5%
